# Prevalence and associated factors of DSM-5 insomnia disorder in the general population of Qatar

**DOI:** 10.1186/s12888-020-03035-8

**Published:** 2021-02-08

**Authors:** Salma Mawfek Khaled, Catalina Petcu, Maryam Ali Al-Thani, Aisha Mohammed H. A. Al-Hamadi, Suhad Daher-Nashif, Monica Zolezzi, Peter Woodruff

**Affiliations:** 1 Social and Economic Survey Research Institute as opposed to Research Institute seperate from Social and Economic Survey, Qatar University, P.O. Box: 2713, Doha, Qatar; 2 College of Medicine, QU-Health and not the other way around, Doha, Qatar; 3 College of Pharmacy, QU-Health, Qatar University, Doha, Qatar; 4 University of Sheffield, Sheffield, UK; 5 Hamad Medical Corporation, Doha, Qatar

**Keywords:** Sleep condition Indicator, DSM-5, Insomnia, Depression, Anxiety, Qatar

## Abstract

**Background:**

Epidemiological studies of insomnia in the Middle East remain scarce. The present study aimed to estimate the prevalence of insomnia and explore its associations in the general population of Qatar. With almost 100 nationalities, Qatar is one of the most culturally diverse, richest, and fastest developing countries in the Arabian Peninsula.

**Methods:**

A probability sample of community-dwelling adults were surveyed in February of 2019. A total of 1611 respondents completed face-to-face interviews in Arabic or English. Logistic regression modeled associations with insomnia, our dependent variable, as defined by a score of ≤16 on the eight-item Sleep Condition Indicator or according to criteria for insomnia in the Diagnostic & Statistical Manual of Mental Disorders, fifth edition or DSM-5.

**Results:**

Approximately, 5.5% of the sample screened positive for insomnia and the 30-day prevalence of those who met all the DSM-5 criteria for insomnia disorder was 3.0%. In addition, 2.0% of the sample screened positive for depression and 3.4% for anxiety in the past 2 weeks. Multivariable analysis showed the following were significantly associated with insomnia: Arab ethnicity, young age, unemployment, being married, having less than high school education, fair or poor health, anxiety, and depression.

**Conclusions:**

Insomnia prevalence was in the lower range of previously reported DSM-defined estimates from developed Western countries. Our findings highlight the need for raising awareness and improving sleep hygiene in potential risk groups such as younger adults and those of Arab ethnicity, in addition to incorporating insomnia screening in the provision of mental health services.

**Supplementary Information:**

The online version contains supplementary material available at 10.1186/s12888-020-03035-8.

## Highlights

- Representative sample of community dwelling adults in Qatar were studied.
- The 30-day prevalence of insomnia disorder was in the range of 3.0–5.5%.
- The prevalence of meeting any of the four DSM-5 criteria ranged between 9.4–13.5%.
- Strong associations between depression, anxiety, and insomnia were observed.
- Insomnia was associated with ethnicity, unemployment, low-education, poor health, and young age.

## Background

According to the Diagnostic and Statistical Manual of Mental Disorders, Fifth edition or DSM-5 [6], insomnia is a sleep disturbance marked by predominant dissatisfaction with sleep quantity or quality that is associated with substantial distress and impairments of daytime functioning. Typical symptoms of insomnia include difficulty initiating sleep, difficulty maintaining sleep (characterized by frequent waking), and early-morning waking with inability to return to sleep [12].

Insomnia adversely affects well-being and quality of life of men and women of all ages and ethnicities [68]. It contributes to considerable healthcare and social challenges, including accidents, impaired social, occupational, educational functioning, and decreased work productivity [60, 68].

To date, most of the research on the epidemiology of insomnia comes from Western European and North American countries [53]. Even so, there are substantial differences in reported findings. These differences may derive from varied sample characteristics, methodologies, cultural contexts, as well as how insomnia is defined. Therefore, it is relevant whether prevalence estimates are based on dissatisfaction with quality or quantity of sleep, symptom-level definitions or international diagnostic criteria. A review conducted by Ohayon in 2002 reported that the highest prevalence estimates (30.0–48.0%) were typical of community-based studies that used the presence of one or more of insomnia symptoms as the defining criteria, while the lowest prevalence estimates (4.4 and 6.4%) were associated with the use of the DSM fourth edition (DSM-IV) diagnostic criteria [53].

Additionally, findings in relation to sociodemographic correlates of insomnia such as gender, age, education, income, and marital status are not always consistent. A study in the United States (US) found that all of these variables were strongly associated with insomnia in the adult population [26]. Similarly, in Spain, gender and age were found to be important variables affecting insomnia with significantly higher prevalence in women than in men and overall prevalence increased with age [55].

From a public health perspective, prevalence estimates are important for service planning, policymaking, and in preventing common sleep disorders like insomnia. In addition, measures of insomnia are important indicators of poor mental health such as impaired concentration, disturbed memory, depression, and anxiety [3, 10, 65]. In fact, insomnia has been recognized as one of the most prevalent symptoms of depression [19, 52, 67] and anxiety disorders [30, 50]. A cohort study conducted in Taiwan which included 19,273 subjects with insomnia and 38,546 subjects without insomnia, showed that respondents with insomnia had a higher risk of developing anxiety, depression or both [14].

Unlike Western countries, the literature on insomnia in the Middle East and most Muslim countries remains sparse. Large-scale epidemiological studies, of prevalence of insomnia and associated clinical features, are scarce. A comprehensive literature review retrieved only one general population study in Turkey. This reported that 15.3% of adults suffer from insomnia [18]. The study investigated the prevalence of sleep disorders in a nationwide representative sample of 5021 Turkish adults. Multiple investigation tools were used to assess the prevalence of sleep disorders. Insomnia was measured by “Yes” response to any of the following, as adapted from DSM–IV–TR: a) difficulty initiating sleep at least three times a week for a month or more; b) difficulty maintaining sleep, a fragmented sleep at least three times a week for a month or more; c) early morning awakening at least once a week in the last month. It was found that insomnia prevalence among women increased with age [18].

Most studies conducted in countries of the Arabian Gulf focus on insomnia in specific populations, such as adolescents and students, or primary care patients. In Kuwait, the rates of insomnia varied between 6.0 to 36.0% in adolescents [1] and between 4.0 to 32.0% in college students [2]. A recent study conducted in primary care centers of one region in Saudi Arabia reported a 60.0% prevalence of insomnia that was significantly higher among 25–40 year-old adults (as well as in those older than 40 years of old) than those younger than 25 years of age and among those having insufficient income [9]. In Qatar, the only study of insomnia reported prevalence as high as 27.0% in a population of soccer players [35]. No study to date estimated the prevalence of insomnia in the general population of Qatar or any of these countries using standard international diagnostic criteria.

With an urban population that continues to grow, Qatar’s context displays a high standard of living and a well-established system of social and healthcare services [7]. The high rate of urban growth, economic development, and a relatively young population [37, 62] as seen in Qatar is associated with the potential risk of insomnia [58] and mental illness [4, 21, 31, 39, 46, 47, 57, 64]. As insomnia is an important risk factor for mental illness [23–25, 44], it is important to determine its prevalence and clinical associations in order to focus effectively on mental health in the population.

Hence, to aid this effort, the present study aimed to estimate the 30-day prevalence of insomnia using DSM-5 criteria and to explore its socio-demographic correlates and associations with depression and anxiety symptoms in the general population of Qatar.

## Methods

This study was part of a larger annual survey conducted in February of 2019 by the Social and Economic Survey Research Institute (SESRI) at Qatar University, which covered various topics related to social, health, economic, and political issues.

### Sample design

As Qatar is divided into eight administrative municipalities, the households in each municipality were stratified according to geographic location and the type of household residents (non-migrants or Qataris, and migrants or resident expatriates hereafter referred to as non-Qataris). The target population included residents of Qatar aged 18 and above who were living in residential units during the survey reference period. For this study, the non-residential units such as army barracks, hospitals, dormitories, camps of blue-collar workers and prisons were excluded. A probability-based sample was then drawn from these strata. As Qataris represented the minority group in the population, they were oversampled. A validated adaptation of the Kish method [38] was utilized to randomly select one eligible adult inside each household in Qatar [43]. Constructed weights accounted for sampling disproportionality and nonresponse.

### Sample size determination

For our survey design, the following formula: *n* = z2(p (1 − p)/e2) deff [16, 38] was used to calculate the required sample size, which was estimated at 1160. The estimate was based on α value of 0.05 or z = 1.96; p or estimate of the proportion set at 50.0% to identify the largest sample size requirement; desired sampling error e = ±3.5%; deff or design effect = 1.48; and a survey response rate = 48.0%. Prior face-to-face surveys conducted by SESRI in the same population have determined the estimates of design effect and survey response rate.

### Language and translation procedures

For the interviews, the measures were available in both English and Arabic, enabling participants to select their preferred language. A team formed of four bilingual research members translated and adapted the measures in the following steps: 1) Each measure was independently translated from English to Arabic by the first author and a researcher; 2) Another two researchers who had not seen the original English version of these measures were assigned to back-translate the two Arabic versions to English; 3) Finally, all team members met and reviewed the two Arabic versions of the questionnaire and compared the original and back-translated English versions. Upon their reviews and comparisons, minor discrepancies in translation arose. These were solved by consensus among team members. Please see *“*Additional file 1*”* for details of survey questions asked in both languages.

### Data collection

Qatar University’s Institutional Review Board approved the study (QU-IRB 339-E/14). The survey questionnaire was programmed in BLAISE and administered using CAPI (Computer Assisted Personal Interview) during face-to-face interviews conducted by trained interviewers in the households of consenting participants who were 18 years old and above. A total of 50 interviewers visited the sampled households and introduced the study. Written informed consent was obtained from all participants after explaining that study participation is voluntary and anonymous. Out of the total sampled households, approximately 4.8% refused to participate. Other reasons for non-participation included language barrier, illness/disability, and vacant housing units. A total of 1611 completed interviews were recorded and the survey response rate was 52.1%. As per household type, the completed interviews included 803 Qataris and 808 non-Qataris. The corresponding maximum sampling error percentage was +/− 3.9 and 3.8 percentage points for Qataris and non-Qataris, respectively.

### Data management

After data collection, all individual interviews were merged and saved in a single BLAISE data file. This dataset was then cleaned, coded, weighted to adjust for probability of selection and non-response, and saved in STATA version 16 for analysis.

### Insomnia

The eight-item Sleep Condition Indicator (SCI) was used to screen for insomnia disorder in the past month based on the DSM-5 criteria [20]. Each item was scored on a 5-point scale (0–4). The scale captured sleep continuity, quality, satisfaction with sleep in addition to symptoms severity, consequences on mood, cognition and day performance and duration of sleep problems. A composite score of all items was computed ranging from 0 to 32 with higher scores indicating better sleep.

As operationalized by the SCI, we measured four main criteria for insomnia disorder as per DSM-5 criteria. The first criterion was difficulty initiating or maintaining sleep. This was based on reports of taking more than 30 min to fall asleep or if waking up during the night, staying awake for greater than 30 min in total; and a rating of sleep quality as average, poor, or very poor. The second criterion was significant distress, which was based on endorsing “somewhat”, “much” or “very much” for the extent that poor sleep in the past month has troubled the respondent in general and endorsing “somewhat”, “much” or “very much” to one of two questions about the extent has poor sleep in the past month affected respondents’ mood, energy, or relationships or affected concentration, productivity, or ability to stay awake. The third criterion was in relation to the frequency of sleep disturbances, which was based on endorsing a minimum of 3 nights per week for the frequency of encountering sleep problems. The fourth criterion was related to the duration of sleep disturbances, based on endorsing a minimum duration of 3 months for having sleep problems.

We estimated 30-day prevalence of insomnia disorder using two methods in relation to the SCI data. First, we used a previously validated cut-off score of 16 or less to denote the proportion of our sample that met minimum criteria for putative insomnia disorder [20]. Second, we used the inherent operationalization of main clinical criteria in the SCI to identify proportion of the sample that met all four main criteria for insomnia disorder.

### Depression

The nine-item Physician Health Questionnaire or PHQ-9 was used to measure the frequency of nine symptoms of depression in the past 2 weeks with a 4-point response including: 0 = “not at all,” 1 = “several days,” 2 = “more than half the days,” and 3 = “nearly every day.” A composite score was calculated based on all nine items and a cut-off score of 10 or higher was used to denote moderate to severe depressive symptoms [41, 59].

### Anxiety

The two-item Generalized Anxiety Disorder (GAD-2) scale was used to measure severity of anxiety symptoms. Although not a diagnostic instrument, the GAD-2 captures the frequency of two symptom criteria for GAD in the DSM-5 including feeling anxious, nervous or on edge and not being able to control or stop worrying over the past 2 weeks. The frequency of these symptoms was captured using the same 4-point response options as describe above for the PHQ-9 [40]. The GAD-2 was scored and a cut-off of 3 or higher was used to denote potentially clinically significant anxiety symptoms [45, 61].

### Covariates

A health-based rating question was used to measure self-reported health status with categories including poor, fair, good, and excellent. Respondents were classified into Qataris or non-Qataris based on self-reported nationality. Ethnicity was determined according to respondent’s country of origin and the language selected to complete the interview. We first coded ethnicity into the following main categories: Arab, South Asian, South East Asian, and other (East Asian, Asian other, African, Latin American, European, from the UK, Russia, US, Canada or Australia). To increase statistical power, we collapsed non-Arab ethnicities into one category to compare with Arab ethnicity. We collected standard socio-demographic information including age, gender, marital status, education level, and employment status.

### Statistical analysis

Weighted proportions, corresponding percentages, and 95% Confidence Intervals (CI) along with raw (unweighted) frequencies (N) were generated to describe the distribution of most of the variables in our sample. Mean and standard deviation (SD) was used to describe age of respondents in years.

Bivariate and multivariable logistic regression analyses were conducted to estimate associations between main independent variables (including depression and anxiety) and insomnia, our main dependent variable. For each bivariate model, we adjusted for the association between one independent variable and insomnia. In the adjusted model, we simultaneously controlled for the associations between all variables and insomnia. Unadjusted (bivariate) and adjusted odds ratios (ORs) with corresponding 95% CI and *p*-values were generated from these models.

## Results

Sample characteristics are shown in Table 1. The sample comprised more females than males and more Arabs than non-Arabs. The respondents were mainly young, non-Qatari adults with post-secondary education. Over half of the sample reported being employed. Less than a tenth rated their current health status as fair or poor. However, only 2.0% of the sample screened positive for depression and 3.4% for anxiety in the past 2 weeks, and 5.5% for insomnia in the past month. The prevalence of those who met all the DSM-5 clinical criteria for insomnia in the past month was 3.0% (Table 1).

**Table 1:** Sample descriptives

	%	CI
**Gender**		
Female	53.2	50.1–56.3
**Marital status**		
Never married	17.5	15.2–20.1
Currently married	79.3	76.6–81.7
Separated/divorced	3.2	2.4–4.3
**Education level**		
Less than high school	10.5	8.9–12.3
High school	21.4	18.9–24.0
Post-secondary	68.1	65.3–70.9
**Arab ethnicity**		
Yes	57.3	54.4–60.1
**Respondent group**		
Qatari household	24.5	23.5–25.6
Non-Qatari household	75.5	74.4–76.5
**Employment status**		
Employed	55.0	51.9–58.1
Unemployed	45.0	41.9–48.1
**Health rating**		
Excellent	45.5	42.4–48.6
Good	46.3	43.2–49.4
Fair	7.4	6.0–9.2
Poor	0.7	0.4–1.6
**General anxiety**		
Yes	3.4	2.5–4.7
**Depression**		
Depressed	2.0	1.4–3.0
**Insomnia**		
Yes	5.5	4.3–7.0
**Insomnia Diagnostic Criteria**		
Yes	3.0	2.1–4.3

Note. % Percentage, CI is 95% confidence Intervals. SD is the standard deviation. All percentages are based on weighted proportions and therefore differ from the raw percentages. General anxiety was measured using the two-item Generalized Anxiety Disorder scale and a cut-off of 3 was used to define moderate-to-severe anxiety symptoms. Depression was measured using the 9-item Physician Health Questionnaire and was defined using a cut-off of 10 to denote moderate-to-severe levels of depression. Insomnia was defined using a cut-off score equal or less than 16 on the Sleep Condition Indicator. Insomnia was also defined using the four main criteria for insomnia disorder as per Diagnostic and Statistical Manual, fifth edition

Table 2 shows that a cut-off score of < 16 (as opposed to > 16 on the SCI) was significantly associated with endorsement on all DSM-5 criteria for insomnia including difficulty initiating or maintaining sleep, significant distress, a minimum of 3 nights per week for frequency of sleep-related disturbances, and a minimum duration of 3 to 6 months for having sleep problem. Also shown in Table 2, the prevalence of meeting any one of the four criteria was higher than meeting all criteria at 10.1% (95% CI: 7.2–13.5), 13.5% (95% CI: 11.5–15.6), 10.3% (95% CI: 8.5–12.1), and 12.2% (95% CI: 10.2–14.2), respectively.

**Table 2:** Pralence of DSM-5 Criteria for those who screen positive for insomnia versus those who do not

Clinical Criteria	Insomnia		No Insomnia		***P***-value	Total Sample	
	%	CI	%	CI		%	CI
**Difficulty initiating or maintaining sleep**^**a**^							
Yes	80.5	71.1–89.8	5.2	3.7–6.7	< 0.001	9.4	7.6–11.2
**Significant distress**^**b**^							
Yes	71.6	59.7–83.4	10.1	8.2–12.0	< 0.001	13.5	11.5–15.6
**Frequency of sleep disturbances**^**c**^							
Yes	86.2	77.9–94.6	5.7	4.2–7.2	< 0.001	10.3	8.5–12.1
**Duration of sleep disturbances**^**d**^							
Yes	86.8	77.8–95.8	7.7	6.0–9.4	< 0.001	12.2	10.2–14.2

Note. % Percentage, CI is 95% confidence Intervals. All percentages are based on weighted proportions and therefore differ from the raw percentages. Clinical criteria was based on DSM-5 as operationalized by the sleep condition indicator. Insomnia is defined by a cut-off score equal or less than 16 on the Sleep Condition Indicator.^a^This criterion is based on report of taking more than 30 min to fall asleep or if wake up during the night will stay awake for greater than 30 min in total and a rating of sleep quality as average, poor, or very poor.^b^This criterion is based on endorsing “somewhat”, “much” or “very much” for the question about the extent that poor sleep in the past month has troubled the respondent in general and endorsing “somewhat”, “much” or “very much” to either questions about the extent has poor sleep in the past month: affected your mood, energy, or relationships or affected concentration, productivity, or ability to stay awake.^c^This criterion is based on endorsing a minimum of 3 nights per week for the frequency of encountering sleep problem.^d^This criterion is based on endorsing a minimum duration of 3 to 6 months for having sleep problem

Table 3 shows age was significantly associated with insomnia in the multivariable model (OR = 0.96, *p* = 0.010); for every unit increase in age, the odds of insomnia decreased by roughly 4.0%. For marital status, the association between never married relative to current married respondents and insomnia became more statistically significant in the multivariable model (Table 3); the odds of insomnia decreased by 62.0% for those who were never married compared to those who were married at the time of the survey.

**Table 3:** Bivariate and Multivariate Logistic Regression Models for Insomnia

	Unadjusted^b^			Adjusted^c^		
	OR	CI	***P***-value	OR	CI	***P***-value
**Gender** (Male ref^a^)						
Female	1.4	0.8–2.4	0.218	0.6	0.3–1.2	0.168
**Age**						
In years	1.0	1.0–1.1	0.198	1.0	0.9–1.0	0.010
**Marital status** (Married ref)						
Never married	0.9	0.5–1.9	0.827	0.4	0.2–0.9	0.027
Separated/divorced	1.7	0.8–3.6	0.193	1.0	0.4–2.5	0.970
**Education level** (Post-secondary ref)						
Less than high school	4.1	2.2–7.8	< 0.001	2.9	1.1–7.6	0.025
High school	1.5	0.8–2.9	0.191	1.2	0.6–2.4	0.518
**Arab ethnicity** (Non-Arab ref)						
Arab	4.8	2.2–10.6	< 0.001	4.5	1.7–11.8	0.002
**Respondent group** (Qataris ref)						
Non-Qataris	0.6	0.4–1.0	0.041	1.3	0.7–2.5	0.461
**Employment status** (Employed ref)						
Unemployed	2.7	1.6–4.5	< 0.001	2.6	1.3–5.1	0.008
**Health rating** (Excellent/good ref)						
Fair/poor	3.2	1.7–6.3	0.001	3.4	1.5–7.9	0.004
**General anxiety** (Non-anxious ref)						
Anxious	7.2	3.5–14.9	< 0.001	3.8	1.7–8.5	0.001
**Depression** (Non-depressed ref)						
Depressed	14.3	6.2–32.6	< 0.001	8.8	3.6–21.4	< 0.001

Note. OR is odds Ratio. CI is 95% confidence intervals. All estimates were weighted. General anxiety was measured using the two-item Generalized Anxiety Disorder scale and a cut-off of 3 was used to define moderate-to-severe anxiety symptoms. Depression was measured using the 9-item Physician Health Questionnaire and was defined using a cut-off of 10 to denote moderate-to-severe levels of depression ^a^Ref represents the reference groups. ^b^Unadjusted model was based on adjustment for only one variable at a time, therefore, n is variable. ^c^Model was based on adjustment for all variables including gender, age, marital status, education, ethnicity, respondent group, employment, health rating, anxiety and depression (*n* = 1468)

In the multivariable analysis, less than high school education, Arab ethnicity, unemployment, fair or poor health rating, anxiety, and depression were independently and significantly associated with insomnia (Table 3). Arab ethnicity increased the odds of reporting insomnia by 350% (OR = 4.50, *p* = 0.002). Having less than excellent or good health rating increased the odds of reporting insomnia by 242% (OR = 3.24, *p* = 0.004). Furthermore, being unemployed increased the odds of reporting insomnia symptoms by 156% (OR = 2.56, *p* = 0.008). The adjusted odds ratio for depression and anxiety symptoms was increased by 777% (OR = 8.77, *p* < 0.001) and 277% (OR = 3.77, *p* = 0.001), respectively, in those with insomnia relative to those without insomnia.

## Discussion

### Prevalence of insomnia

To our knowledge, this is the first report on prevalence of insomnia disorder and associated clinical factors in a representative sample of community dwelling adults in the state of Qatar. Our study makes an important contribution as most of the published studies from the Middle East to date have rarely used a standard set of international diagnostic criteria to estimate the burden of insomnia in the general population. Overall, our prevalence estimates ranged between 3.0% using the DSM-5 diagnostic criteria or 5.5% using the SCI cut-off score to define insomnia. Our estimates were relatively lower than those reported in developed Western countries [54]. However, comparisons between the prevalence estimates from our study and these should only be made with caution. For example, a pooled prevalence estimate of 9.8% was reported in seven European countries (as defined by meeting all of the DSM-IV criteria for insomnia disorder- including the presence of a predominant complaint of insomnia, lasting for at least 1 month, that caused significant distress or daytime consequences) [54]. The lower prevalence estimate of 5.5% in our study may stem from differences in criteria especially that the DSM-5 criteria stipulate minimum of 3 months for chronicity of insomnia symptoms as opposed to minimum of 1 month in the DSM-IV. Additionally, our time frame for assessing prevalence of insomnia was 1 month only compared to a minimum of 1 month or more in the other studies [54]. However, our estimates ranged from 9.4 to 13.5%, and were similar to that reported in the general population of Turkey, where the prevalence of meeting any DSM-IV-TR criteria was estimated at 15.3% [18].

### Socio-demographic factors

In our study increase in age was associated with decrease in insomnia symptoms in contrast to most worldwide reports that highlight a higher prevalence of insomnia among older populations [22, 53, 56, 63]. A meta-analysis of general population studies in China found that younger participants were more likely to suffer from insomnia than older participants. This finding was attributed to higher daily stress of modern urban life and high technology media use [13]. Similar factors may explain the higher prevalence of insomnia in younger respondents in Qatar who are mostly adult migrants and Qatari nationals between the ages of 25–54 years comprising 70% of the country’s population [33]. An additional explanation of why insomnia symptoms may decrease with older age is that in Arab Muslim societies, practicing filial piety and honoring parents is a religious obligation and a social norm [66], and most of the elderly live close to at least one son or a daughter. This family support may account for lower levels of daily stress and consequently insomnia amongst older adults in Arab-Muslim societies.

### Insomnia and mental illness

Overall, findings with respect to strong associations between insomnia and depression and between insomnia and anxiety in our study population are in line with findings from around the world [3, 10, 19, 30, 50, 52, 65, 67].

These findings also reinforce our suggestion that there is a need for Public Health consideration to address insomnia symptoms early, particularly due to stigma association with mental disorders. The association between insomnia and mental illness may have important implications in the overall help-seeking and health management of individuals with symptoms of insomnia. Furthermore, considering the potential clinical importance of insomnia, it is worthwhile to include insomnia-related questions in depression and anxiety screening questionnaires.

### Cultural considerations

Arab ethnicity was positively associated with insomnia in our study. Arabs reported poorer sleep continuity, severity, quality and daytime impact of poor sleep than non-Arabs. Cultural factors are likely to explain these results. Late bedtimes, interrupted sleep, and daytime napping have been reported in studies investigating sleeping patterns among Middle Eastern populations, factors that may result in suboptimal sleep durations and quality of sleep [36, 49]. Extreme hot temperatures in the daytime in the Arabian Gulf often lead to an increase in nighttime socialization and late bedtimes. In addition, religious obligations such as daybreak prayer, Sohour in the fasting month, Ramadan, require interruption of sleep and changes in eating habits. Although there is scarce scientific information on how the effects of adhering to religious teachings affect sleep and health, it is generally well established that uninterrupted sleep, particularly in the initial stages of sleep, enhances sleep quality [11]. Our results indicate that these patterns of sleep, which are usually embedded in the Arab-Muslim culture, may put them at risk of insomnia. Our findings are also consistent with available literature on the role of culture in shaping beliefs, attitudes, and practices about sleep [29] as well as cross-cultural adjustment to sleep pattern changes [28].

International guidelines for insomnia management recommend the provision of patient education of healthy sleep habits known as sleep hygiene. Sleep hygiene consists of a combination of behavioral practices and environmental conditions, which improve sleep [5]. The results of this study highlight the need for public awareness campaigns focused on sleep hygiene and optimal lifestyle habits that would help improve sleep quality and reduce insomnia symptoms in this population. A meta-analysis study assessing the efficacy of sleep hygiene education for insomnia found a significant improvement in sleep onset latency, sleep fragmentation, total sleep duration and insomnia scores indicative of improved sleep [15].

Another important finding in our study was that unemployment was positively associated with insomnia. Similar findings were reported in several studies around the world, which showed that people with higher socioeconomic status tend to have less symptoms of insomnia and better quality of sleep [17, 42]. This finding is understandable, as unemployment may be associated with stress related to losing residency status in the country and fear of financial difficulties for non-Qataris. In addition, it has been reported that unemployment can lead to symptoms of depression [48]. As such, three key determinants of insomnia in present study, such as unemployment, depression and anxiety, appear to be interconnected.

### Limitations

The survey was cross-sectional and based on self-report. Although survey questionnaires were thoroughly translated and administered in the mother tongue of most respondents, we cannot rule out other sources of response bias in Arab and non-Arab migrants. However, we tried to mitigate the potential risk of response bias (e.g. social desirability) by training all interviewers in and systematically monitoring their adherence to standardized survey interviewing techniques. We collected no information about lifestyle factors that may be associated with insomnia and mental health such as cigarette smoking [32, 34] and diet [8, 27, 70] or shift or night work [51, 69], which is a limitation of the current analysis. The SCI is a relatively new tool for screening for insomnia according to the DSM-5 criteria and its psychometric properties in Arabic speaking populations have not previously determined. Differential diagnosis or causes by organic medical diseases as required by the DSM-5 criteria were not verified. Additionally, clinical evaluation, the gold standard for establishing insomnia diagnosis, was not carried out in this study. It is recommended that future studies establish the clinical validity of the SCI in the context of Arabic speaking populations.

## Conclusions

This is the first large epidemiological study conducted in the general population in Qatar about the prevalence of insomnia and associated factors. The study suggests that there is an association between mental illness (particularly depression and anxiety) and insomnia, that needs to be taken into account particularly when screening individuals for mental illness and in the provision of mental health services. We also report important findings with regard to at-risk age groups and ethnicity that highlight the need for raising awareness of sleep hygiene in these groups.

This study suggests the need for further research into the socio-cultural and political factors affecting insomnia prevalence in Arab populations. Given the time of this publication, we also suggest conducting a similar study during COVID-19 era, in order to measure its impact on insomnia prevalence. Our study can provide a baseline prevalence of insomnia in the Arabian Gulf Region against which other studies can be compared, and to aid assessment of the impact of natural, political and social crises on insomnia and mental health.

## Supplementary Information

**Additional file 1.** Survey Questionnaire in English and Arabic

## Data Availability

The datasets used and/or analysed during the study are available from the corresponding author on reasonable request.

## Abbreviations

SCI: Sleep Condition Indicator
DSM-5: Diagnostic and Statistical Manual of Mental Disorders - 5th Edition
PHQ-9: Physician Health Questionnaire-9
GAD-2: Generalized Anxiety Questionnaire-2
CI: Confidence interval
OR: Odds ratio
SD: Standard deviation
