# Supplementary material for: Prevalence and associated factors of DSM-5 insomnia disorder in the general population of Qatar
Source: BMC Psychiatry. 2021 Feb 8;21:84. doi: 10.1186/s12888-020-03035-8 (PMC7869071; doi:10.1186/s12888-020-03035-8)
Supplement: Supplementary file 1 — Additional file 1. Survey Questionnaire in English and Arabic [file 12888_2020_3035_MOESM1_ESM.pdf]

Appendix - Survey Questionnaires

1. Gender

INTERVIEWER: enter the gender of the respondent. Don't ask for it

1. Male
2. Female

2. Are you never married, currently married, separated, divorced, or widowed?

1 Never married

2 Currently married

3 Separated

4 Divorced

5 Widowed

6 DON'T KNOW

7 REFUSED

3. What is your nationality? **[PROGRAMMER: USE NUMERIC LIST OF COUNTRIES]**

4. In what year were you born? ___________

5. What is the highest level of education you have completed? [INTERVIEWER: PROBE FROM CATEGORIES IF NECESSARY]

1. NEVER ATTENDED ANY SCHOOL
2. PRIMARY (1-6 years)
3. PREPARATORY (7-9 years)
4. Vocational (after PREPARATORY but no secondary)
5. Secondary (10-12)
6. Diploma (after secondary but no university)
7. University graduate/BA/BCom/BSc

8. Master’s degree
9. Ph.D.
10. OTHER (SPECIFY)

98 DON’T KNOW

99 REFUSED

6. Are you currently employed?

1 YES

2 NO

8 DON’T KNOW

9 REFUSED

7. What do you consider you ethnic origin?

1. Arab

2. Indian

3. Pakistani

4. Bangladeshi

5. Nepali

6. Filipino

6. Asian (other specify)

7. Persian/Iranian

8. Turkish

9. African

10. American

11. British

12. European

13. Latino/South American

14. Other (specify)

8. How do you rate your overall health? Would you say it is…?

1) Excellent

2) Good

3) Fair

4) Poor

89 DON'T KNOW

99 REFUSE

Sleep Condition Indicator

The following questions are about your sleep

Item	Score				
	4	3	2	1	0
Thinking about a typical night in the last month…					
…..how long does it take you to fall asleep?	0–15 min	16–30 min	31–45 min	46–60 min	≥ 61 min
….if you wake up during the night, how long are you awake for in total (add all the wakenings up)	0–15 min	16–30 min	31–45 min	46–60 min	≥ 61 min
….how many nights a week do you have a problem with your sleep?	0–1	2	3	4	5–7
….how would you rate your sleep quality?	Very good	Good	Average	Poor	Very poor
Thinking about the past month, to what extent has poor sleep…					
….affected your mood, energy, or relationships?	Not at all	A little	Some-what	Much	Very much
….affected your concentration, productivity, or ability to stay awake?	Not at all	A little	Some-what	Much	Very much
….troubled you in general?	Not at all	A little	Some-what	Much	Very much
Finally…					

….how long have you had a problem with your sleep?	I don't have a problem or I have problem with sleep for less than 1 month	1–2 months	3–6 months	7–12 months	more than one year

Physician Health Questionnaire (PHQ-9)

Over the last two weeks, how often have you been bothered by any of the following problems?

Reduced interest or pleasure in doing things? Would you say….

1. Not at all
2. Several days
3. More than half the days
4. Nearly every day

89DON'T KNOW

99REFUSE

Feeling down, depressed, sad or hopeless? Would you say….

1. Not at all
2. Several days
3. More than half the days
4. Nearly every day

89 DON'T KNOW

99 REFUSE

Trouble falling or staying asleep or sleeping too much? Would you say….

1. Not at all
2. Several days
3. More than half the days
4. Nearly every day

89 DON'T KNOW

99 REFUSE

Feeling tired or having little energy?

1. Not at all
2. Several days
3. More than half the days
4. Nearly every day

89 DON'T KNOW

99 REFUSE

Poor appetite or overeating?

1. Not at all
2. Several days
3. More than half the days
4. Nearly every day

89 DON'T KNOW

99 REFUSE

Feeling bad about yourself — or that you are a failure or have let yourself or your family down?

1. Not at all
2. Several days
3. More than half the days
4. Nearly every day

89 DON'T KNOW

99 REFUSE

Trouble concentrating on things, such as reading the newspaper or watching television?

1. Not at all

2. Several days

3. More than half the days

4. Nearly every day

89 DON'T KNOW

99 REFUSE

Moving or speaking so slowly that other people could have noticed? Or the opposite — being so fidgety or restless that you have been moving around a lot more than usual?

1. Not at all

2. Several days

3. More than half the days

4. Nearly every day

89 DON'T KNOW

99 REFUSE

Thoughts that you would be better off dead or of hurting yourself in some way

1. Not at all

2. Several days

3. More than half the days

4. Nearly every day

89 DON'T KNOW

99 REFUSE

IV, READ ONLY IF RESPONDENT UPSET OR ANGRY THAT WE ASK THIS

QUESTION: My apologies. We ask these questions because research shows they do apply to some people and to treat everyone the same am required to read it to all. Any question you do not wish to answer just let me know and we can skip it.

Generalized Anxiety Disorder (GAD-2)

Over the last two weeks, how often have you been bothered by any of the following problems?

Feeling nervous, anxious or on edge

1. Not at all

2. Several days

3. More than half the days

4. Nearly every day

89 DON'T KNOW

99 REFUSE

Not being able to stop or control worrying

1. Not at all

2. Several days

3. More than half the days

4. Nearly every day

89 DON'T KNOW

99 REFUSE

الأسئلة المشتركة

1. (أدخل جنس المستجيب) الباحث: أدخل نوع المشترك. لا تسأل عنه

1 ذكر

2 أنثى

لا أعرف/ لا يمكنني التعرف على ذلك 8
9

2. ماهي حالتك الاجتماعية؟ هل أنت متزوج، منفصل، مطلق، أرمل، لم تتزوج مطلقاً؟

1. لم يسبق لي الزواج
2. متزوج حاليًا
3. منفصل
4. مطلق
5. أرمل
8. لا أعرف
9. أرفض الإجابة

3. ما هي جنسيتك؟

[PROGRAMMER: USE NUMERIC LIST OF COUNTRIES]

4. في أي عام ولدت؟ ________________

5. ما هو أعلى مستوى تعليمي أكملته؟

1. لم أذهب إلى المدرسة على الإطلاق
2. الابتدائي (1-6)
3. الإعدادي (7-9)
4. الثانوي (10-12)
5. تدريب مهني بعد الثانوية أو دبلوم (برنامج أقل من سنتين)
6. درجة مشارك/معهد متوسط (برنامج سنتين بعد الثانوية)
7. شهادة بكالوريوس

8. شهادة ماجستير

9. شهادة دكتوراه (دكتوراه فلسفية، دكتوراه تعليمية)

10. غير ذلك (حدد)

98. لا أعرف

99. أرفض الإجابة

6. هل تعمل حاليًا؟

1 نعم

2 لا

8 لا أعرف

9 أرفض الإجابة

7. ما هو انتمائك العرقي؟

1. عربي
2. هندي
3. باكستاني
4. بنغلاديشي
5. النيبالية
6. الفلبينية
7. آسيا (أخرى، تحديد)
8. فارسي / إيراني
9. تركي
10. افريقي
11. امريكي
12. بريطاني
13. أوروبي
14. لاتيني / من أمريكا الجنوبية
15. غير ذلك (يرجى التحديد)

8. كيف تقيم صحتك بشكل عام؟ هل ترى أنها ...؟

1. ممتازة
2. جيدة
3. مقبولة
4. سيئة
8. لا أعرف
9. أرفض الإجابة

مؤشر حالة النوم

الأسئلة التالية تتعلق بنومك

العناصر	الدرجات				
	4	3	2	1	0
فكر بليلة عادية خلال الشهر الماضي ...					
.... كم تستغرق من الوقت قبل الخلود إلى النوم ؟	15-0 دقيقة	30-16 دقيقة	45-31 دقيقة	60-46 دقيقة	≤ 61 دقيقة
.... إذا استيقظت اثناء الليل، لكم من الوقت تبقى مستيقظ؟ (المجموع الكلي لفترات الاستيقاظ)	15-0 دقيقة	30-16 دقيقة	45-31 دقيقة	60-46 دقيقة	≤ 61 دقيقة
... كم ليلة في الأسبوع واجهت خلالها مشاكل وأنت تحاول أن تنام؟	1-0	2	3	4	7-5
... كيف تقيم جودة نومك؟	جيدة جداً	جيدة	متوسطة	سيئة	سيئة جداً
بالنظر إلى الماضي ...					
... إلى أي مدى أثر سوء النوم على مزاجك، أو طاقتك، أو علاقاتك؟	أبدا	قليلاً	نوعا ما	كثيراً	كثيراً جداً
... إلى أي مدى أثر سوء النوم على تركيزك، أو انتاجيتك / تحصيلك، أو قدرتك على البقاء مستيقظا؟	أبدا	قليلاً	نوعا ما	كثيراً	كثيراً جداً
... إلى أي مدى أثر سوء النوم عليك بشكل عام ؟	أبدا	قليلاً	نوعا ما	كثيراً	كثيراً جداً
أخيراً...					

.... منذ متى وأنت تعاني من مشاكل النوم؟	ليس لدي مشاكل في أوعانيت النوم من مشاكل في لفترة النوم اقل من شهر	1-2 أشهر	3-6 أشهر	7-12 أشهر	أكثر من سنة

استبيان حول صحة المريض

خلال الأسبوعين الماضيين، كم مرة عانيت من أي من المشاكل التالية؟

قلة الإهتمام أو قلة الإستمتاع بممارسة أو القيام بأي عمل.

1. ولا مرة
2. عدة أيام
3. أكثر من نصف الأيام
4. تقريباً كل يوم
8. لا أعرف
9. أرفض الإجابة

الشعور بالحزن أو ضيق الصدر أو اليأس

1. ولا مرة
2. عدة أيام
3. أكثر من نصف الأيام
4. تقريباً كل يوم
10. لا أعرف
11. أرفض الإجابة

صعوبة في النوم أو نوم مقطع أو النوم أكثر من المعتاد

1. ولا مرة
2. عدة أيام
3. أكثر من نصف الأيام
4. تقريباً كل يوم
8. لا أعرف

9. أرفض الإجابة

الشعور بالتعب أو بإمتلاك القليل جداً من الطاقة

1. ولا مرة
2. عدة أيام
3. أكثر من نصف الأيام
4. تقريباً كل يوم
8. لا أعرف
9. أرفض الإجابة

الزيادة في تناول الطعام عن المعتاد قلة الشهية او

1. ولا مرة
2. عدة أيام
3. أكثر من نصف الأيام
4. تقريباً كل يوم
8. لا أعرف
9. أرفض الإجابة

الشعور بعدم الرضا عن النفس أو الشعور بأنك قد خذلت نفسك أو عائلتك

1. ولا مرة
2. عدة أيام
3. أكثر من نصف الأيام
4. تقريباً كل يوم
8. لا أعرف
9. أرفض الإجابة

صعوبة في التركيز مثلاً أثناء قراءة الصحيفة أو مشاهدة التلفزيون

1. ولا مرة
2. عدة أيام
3. أكثر من نصف الأيام

4. تقريباً كل يوم
8. لا أعرف
9. أرفض الإجابة

بطء في الحركة أو بطء في التحدث عما هو معتاد لدرجة ملحوظة من الآخرين/ أو على العكس من ذلك التحدث بسرعة وكثرة الحركة أكثر من المعتاد

1. ولا مرة
2. عدة أيام
3. أكثر من نصف الأيام
4. تقريباً كل يوم
8. لا أعرف
9. أرفض الإجابة

راودتك أفكار بأنه من الأفضل لو كنت ميت(ة) أو أفكار بأن تقوم(ي) بإيذاء

1. ولا مرة
2. عدة أيام
3. أكثر من نصف الأيام
4. تقريباً كل يوم
8. لا أعرف
9. أرفض الإجابة

مجري المقابلة، إقرأ فقط في حال انزعج المستجيب او غضب لأننا قمنا بسؤاله هذا السؤال: اعتذر. نحن نسأل هذه الأسئلة لأن البحث يبين أنها تنطبق على بعض الناس ولمعاملة الجميع بنفس الطريقة علي قراءة كل الأسئلة للجميع. أعلمني بخصوص أي سؤال لا ترغب بالإجابة عنه ويمكننا تخطيه.

مقياس اضطراب القلق العام

خلال الأسبوعين الماضيين، كم مرة عانيت من أي من المشاكل التالية؟

الشعور بالغضب أو القلق أو الانفعال الشديد

1. ولا مرة
2. عدة أيام
3. أكثر من نصف الأيام

4. تقريباً كل يوم

8. لا أعرف

9. أرفض الإجابة

عدم القدرة على إنهاء القلق أو التحكّم فيه

1. ولا مرة

2. عدة أيام

3. أكثر من نصف الأيام

4. تقريباً كل يوم

8. لا أعرف

9. أرفض الإجابة
